# Supplementary material for: Transposable elements contribute substantially to naturally occurring genetic lethality in Drosophila melanogaster
Source: PLoS Biol. 2026 Mar 10;24(3):e3003467. doi: 10.1371/journal.pbio.3003467 (PMC12974806; doi:10.1371/journal.pbio.3003467)
Supplement: S1 Table — (DOCX) [file pbio.3003467.s001.docx]

# **S1 Table: Deficiency lines used for coarse-mapping**

| Arm | Deficiency ID | start | end | RRID |
| --- | --- | --- | --- | --- |
| L | DF(2L)H2O | 16692000 | 18359026 | RRID:BDSC_3180 |
| L | DF(2L)r10 | 15476957 | 16549467 | RRID:BDSC_1491 |
| L | DF(2L)ED775 | 12010010 | 12975028 | RRID:BDSC_8907 |
| L | DF(2L)ED1303 | 19517610 | 20382385 | RRID:BDSC_8679 |
| L | DF(2L)ED1315 | 20085397 | 20917519 | RRID:BDSC_9269 |
| L | DF(2L)ED793 | 13934848 | 14689337 | RRID:BDSC_9061 |
| L | DF(2L)J39 | 10272324 | 11013206 | RRID:BDSC_1469 |
| L | DF(2L)ED761 | 11808835 | 12436439 | RRID:BDSC_24109 |
| L | DF(2L)ED678 | 8958155 | 9581740 | RRID:BDSC_8906 |
| L | DF(2L)ED1272 | 19158440 | 19753324 | RRID:BDSC_24116 |
| L | DF(2L)ED1473 | 21250892 | 21828548 | RRID:BDSC_9266 |
| L | DF(2L)ED1378 | 20823195 | 21397328 | RRID:BDSC_9682 |
| L | DF(2L)C' | 22943135 | 23515712 | RRID:BDSC_4959 |
| L | DF(2L)BSC244 | 11445733 | 12002748 | RRID:BDSC_9718 |
| L | DF(2L)ED1203 | 18617225 | 19158447 | RRID:BDSC_8935 |
| L | DF(2L)ED7762 | 1657408 | 2197121 | RRID:BDSC_24119 |
| L | DF(2L)BSC233 | 7388190 | 7887564 | RRID:BDSC_9708 |
| L | DF(2L)BSC204 | 8529124 | 9025734 | RRID:BDSC_9631 |
| L | DF(2L)ED385 | 5980272 | 6465772 | RRID:BDSC_9341 |
| L | DF(2L)ED690 | 9437469 | 9918174 | RRID:BDSC_24133 |
| L | DF(2L)ED94 | 568095 | 1036969 | RRID:BDSC_8908 |
| L | DF(2L)BSC691 | 13512897 | 13721648 | RRID:BDSC_26543 |
| L | DF(2L)lt109 | 22536884 | 22981816 | RRID:BDSC_26782 |
| L | DF(2L)BSC241 | 11006679 | 11445740 | RRID:BDSC_9716 |
| L | DF(2L)BSC277 | 12832803 | 13257491 | RRID:BDSC_23662 |
| L | DF(2L)BSC295 | 4031318 | 4455780 | RRID:BDSC_23680 |
| L | DF(2L)ed1 | 3619097 | 4031377 | RRID:BDSC_5330 |
| L | DF(2L)ED441 | 6709099 | 7084679 | RRID:BDSC_24652 |
| L | DF(2L)ED489 | 7204186 | 7576637 | RRID:BDSC_24127 |
| L | DF(2L)ED250 | 4477085 | 4821294 | RRID:BDSC_9270 |
| L | DF(2L)ED334 | 5658629 | 5999667 | RRID:BDSC_9343 |
| L | DF(2L)ED1102 | 16350236 | 16684883 | RRID:BDSC_24113 |
| L | DF(2L)Exel8038 | 18123514 | 18455586 | RRID:BDSC_7840 |
| L | DF(2L)Exel6008 | 2494660 | 2755377 | RRID:BDSC_7494 |
| L | DF(2L)Exel6030 | 11807409 | 11971081 | RRID:BDSC_7513 |
| L | DF(2L)BSC323 | 11870517 | 12055450 | RRID:BDSC_24348 |
| L | DF(2L)ED50001 | 0 | 72671 | RRID:BDSC_24626 |
| R | DF(2R)ED3683 | 18288869 | 19228991 | RRID:BDSC_95324 |
| R | DF(2R)BSC595 | 10385967 | 11288578 | RRID:BDSC_25428 |
| R | DF(2R)ED1612 | 6220532 | 7049672 | RRID:BDSC_8045 |
| R | DF(2R)BSC597 | 21806350 | 22592996 | RRID:BDSC_25430 |
| R | DF(2R)BSC630 | 5127919 | 5782413 | RRID:BDSC_25705 |
| R | DF(2R)BSC594 | 19632024 | 20199035 | RRID:BDSC_25678 |
| R | DF(2R)ED1715 | 7326951 | 7916923 | RRID:BDSC_8931 |
| R | DF(2R)ED3610 | 17850905 | 18412033 | RRID:BDSC_9066 |
| R | DF(2R)ED3791 | 20698411 | 21250981 | RRID:BDSC_9267 |
| R | DF(2R)ED1742 | 8174168 | 8724129 | RRID:BDSC_9276 |
| R | DF(2R)ED1673 | 6985802 | 7533553 | RRID:BDSC_9062 |
| R | DF(2R)ED1725 | 7613924 | 8156045 | RRID:BDSC_8941 |
| R | DF(2R)ED2426 | 15128808 | 15610824 | RRID:BDSC_9064 |
| R | DF(2R)BSC780 | 24203216 | 24685191 | RRID:BDSC_27352 |
| R | DF(2R)ED2219 | 11197412 | 11665391 | RRID:BDSC_8910 |
| R | DF(2R)BSC702 | 20424117 | 20870855 | RRID:BDSC_26554 |
| R | DF(2R)BSC769 | 22940931 | 23385467 | RRID:BDSC_26866 |
| R | DF(2R)BSC604 | 24532173 | 24968432 | RRID:BDSC_25437 |
| R | DF(2R)BSC889 | 5782239 | 6181745 | RRID:BDSC_32253 |
| R | DF(2R)BSC880 | 12496830 | 12894106 | RRID:BDSC_30585 |
| R | DF(2R)BSC136 | 23666959 | 24057464 | RRID:BDSC_9424 |
| R | DF(2R)ED2247 | 11600106 | 11988720 | RRID:BDSC_8912 |
| R | DF(2R)BSC701 | 20278834 | 20667273 | RRID:BDSC_26553 |
| R | DF(2R)BSC427 | 15534718 | 15922119 | RRID:BDSC_24931 |
| R | DF(2R)BSC308 | 15680216 | 16031279 | RRID:BDSC_23691 |
| R | DF(2R)BSC281 | 10125229 | 10462874 | RRID:BDSC_23666 |
| R | DF(2R)X1 | 9927457 | 10249435 | RRID:BDSC_1702 |
| R | DF(2R)BSC273 | 13159579 | 13502150 | RRID:BDSC_23169 |
| R | DF(2R)BSC307 | 13623008 | 13961601 | RRID:BDSC_23690 |
| R | DF(2R)BSC280 | 9292659 | 9578616 | RRID:BDSC_23665 |
| R | DF(2R)BSC360 | 21497210 | 21970239 | RRID:BDSC_24384 |
| R | DF(2R)ED2308 | 12780370 | 12996984 | RRID:BDSC_9268 |
| R | Df(2R)M41A10 | 1 | 5259004 | RRID:BDSC_741 |
