# Supplementary material for: Transposable elements contribute substantially to naturally occurring genetic lethality in Drosophila melanogaster
Source: PLoS Biol. 2026 Mar 10;24(3):e3003467. doi: 10.1371/journal.pbio.3003467 (PMC12974806; doi:10.1371/journal.pbio.3003467)
Supplement: S2 Table — (DOCX) [file pbio.3003467.s002.docx]

**S2 Table: Fine-mapping deficiency lines**

| Arm | Deficiency ID | start | end | RRID |
| --- | --- | --- | --- | --- |
| R | DF(2R)BSC265 | 7,782,827 | 7,939,047 | RRID:BDSC_23164 |
| R | DF(2R)BSC266 | 7,997,576 | 8,462,638 | RRID:BDSC_26500 |
| R | DF(2R)BSC697 | 5,674,498 | 5,782,429 | RRID:BDSC_26549 |
| R | DF(2R)BSC696 | 5,417,100 | 5,782,429 | RRID:BDSC_26548 |
| R | DF(2R)Exel7128 | 13,801,956 | 13,897,827 | RRID:BDSC_7873 |
| R | DF(2R)50C102 | 13,954,125 | 13,964,325 | RRID:BDSC_8111 |
| R | DF(2R)BSC383 | 13,839,479 | 14,024,879 | RRID:BDSC_24407 |
| L | DF(2L)ED5878 | 67,365 | 161,120 | RRID:BDSC_9353 |
| L | DF(2L)ED354 | 5,980,272 | 6,083,233 | RRID:BDSC_9187 |
| L | DF(2L)BSC239 | 6,079,298 | 6,197,305 | RRID:BDSC_9714 |
| L | DF(2L)BSC185 | 6,127,717 | 6,465,713 | RRID:BDSC_9613 |
| L | DF(2L)BSC781 | 16,325,113 | 16,417,726 | RRID:BDSC_27353 |
| L | DF(2L)Exel7063 | 15,426,051 | 15,744,445 | RRID:BDSC_7831 |
| L | DF(2L)ED7007 | 6,709,099 | 6,963,808 | RRID:BDSC_8936 |
| L | DF(2L)ED6569 | 6,709,099 | 6,921,292 | RRID:BDSC_8940 |
| L | DF(2L)BSC108 | 6,874,407 | 6,945,223 | RRID:BDSC_8847 |
| R | DF(2R)BSC879 | 11,892,100 | 12,142,362 | RRID:BDSC_30584 |
| R | DF(2R)BSC259 | 11,593,011 | 11,823,771 | RRID:BDSC_23159 |
