# Supplementary material for: Transposable elements contribute substantially to naturally occurring genetic lethality in Drosophila melanogaster
Source: PLoS Biol. 2026 Mar 10;24(3):e3003467. doi: 10.1371/journal.pbio.3003467 (PMC12974806; doi:10.1371/journal.pbio.3003467)
Supplement: S3 Table — (DOCX) [file pbio.3003467.s003.docx]

**S3 Table: Gene knock-out lines**

| Arm | Gene | RRID |
| --- | --- | --- |
| R | drosha | RRID:BDSC_92831 |
| R | Nipped-A | RRID:BDSC_16514 |
| R | Nipped-A | RRID:BDSC_7188 |
| R | Mitochondrial trans-2-enoyl-CoA reductase (Mecr) | RRID:BDSC_92694 |
| R | tumbleweed (tum) | RRID:BDSC_8687 |
| R | short stop (shot) | RRID:BDSC_10522 |
| L | lethal (2) giant larvae (l(2)gl) | Obtained from the Lai Lab at Sloan Kettering Institute |
| L | Kr transcription factor homolog 1 (Kr-h1) | RRID:BDSC_10381 |
| L | beaten path Ia | RRID:BDSC_4742 |
| L | Bicaudal C (BicC) | RRID:BDSC_30473 |
| L | Ca2+-channel protein α1 subunit D (Ca-α1D) | RRID:BDSC_13282 |
| L | Fibroblast growth factor receptor 1 oncogene partner 2 (Fgop2) | RRID:BDSC_15100 |
| L | Heterogeneous nuclear ribonucleoprotein at 27C (Hrb27C) | RRID:BDSC_10375 |
| L | CG18304 | RRID:BDSC_13554 |
| R | Suppressor of zeste 2 (Su(z)2) | RRID:BDSC_5548 |
| R | Posterior sex combs (Psc) | RRID:BDSC_5547 |
