# Supplementary material for: Transposable elements contribute substantially to naturally occurring genetic lethality in Drosophila melanogaster
Source: PLoS Biol. 2026 Mar 10;24(3):e3003467. doi: 10.1371/journal.pbio.3003467 (PMC12974806; doi:10.1371/journal.pbio.3003467)

# **S1 Fig: Integrative Genomics Viewer (IGV) Phased Haplotypes of Telomeric Deletions**

Wild type (complemented with our *l(2)gl* knock-out)


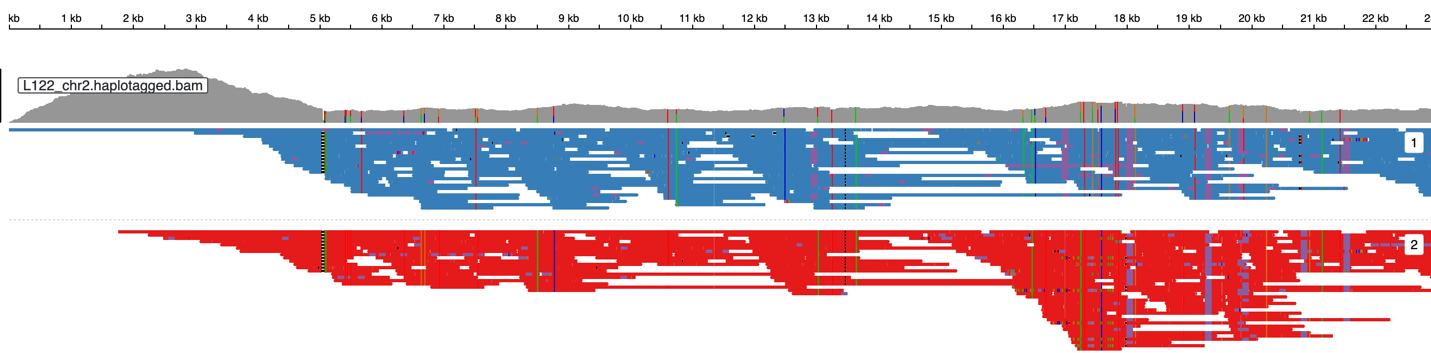


The following sublines did not complement with *l(2)gl.*


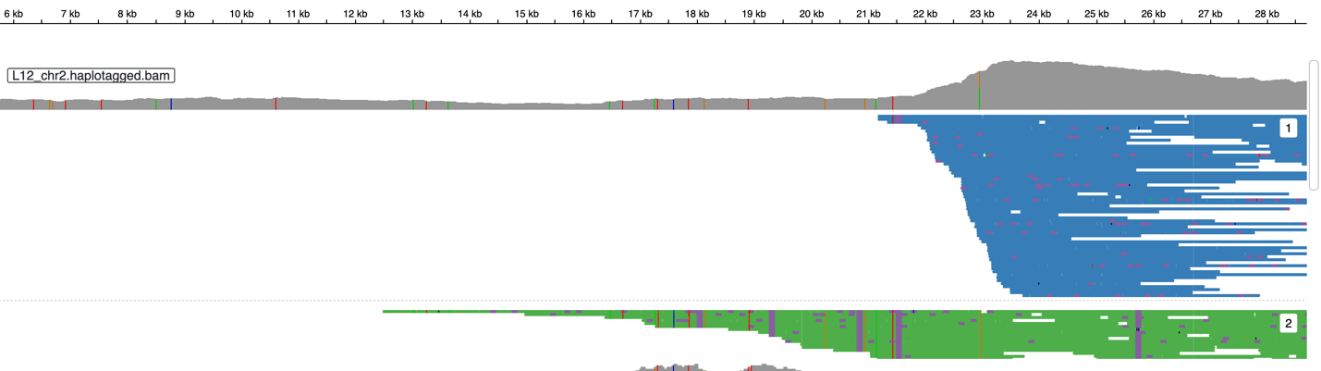


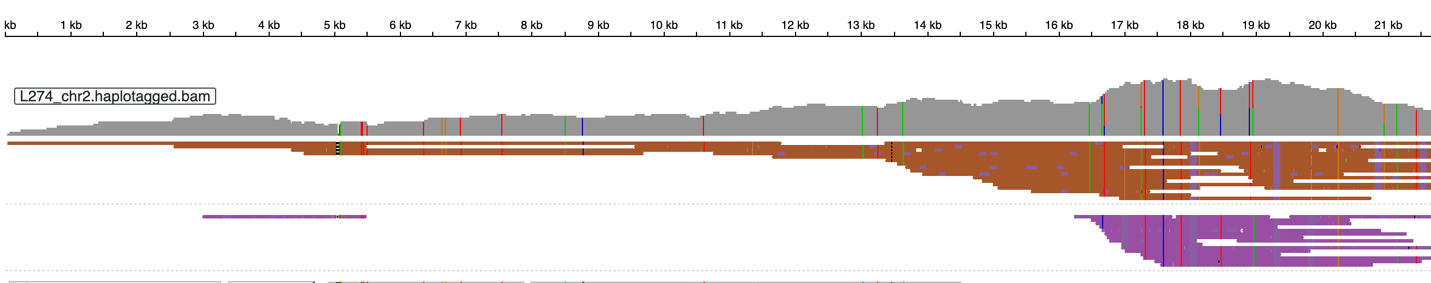


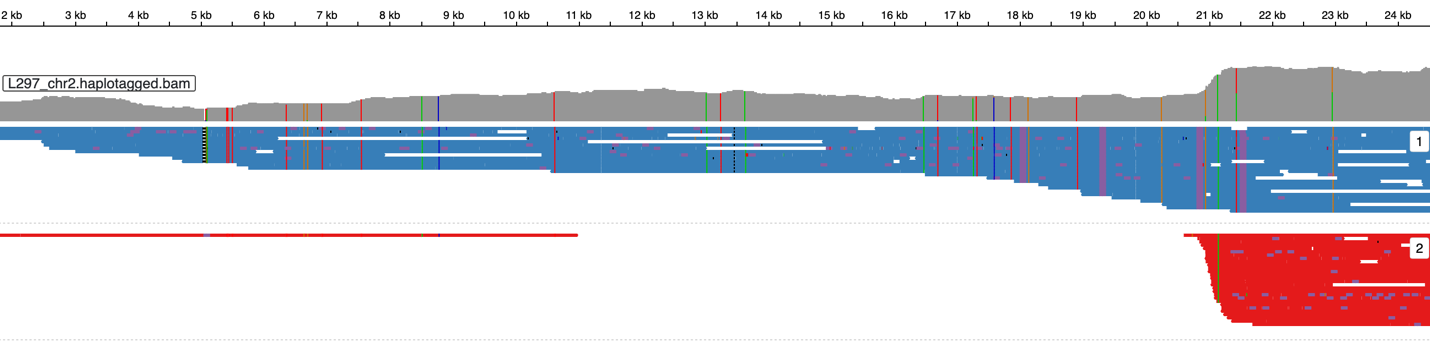


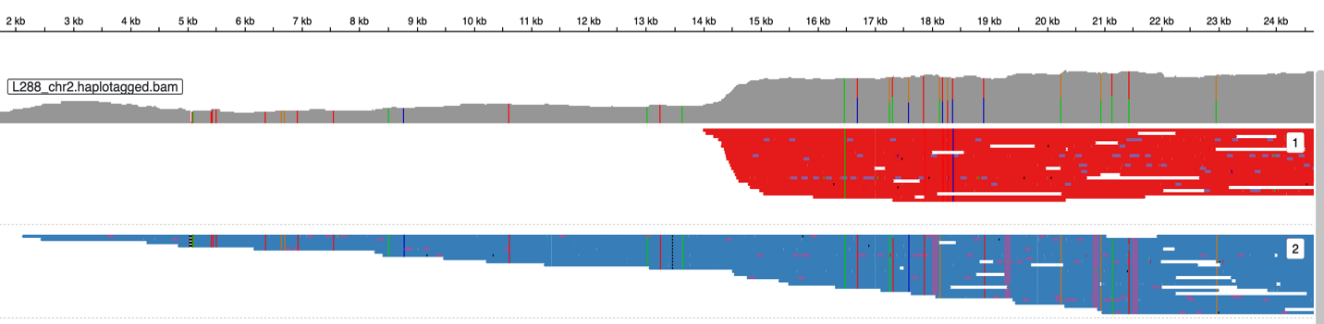


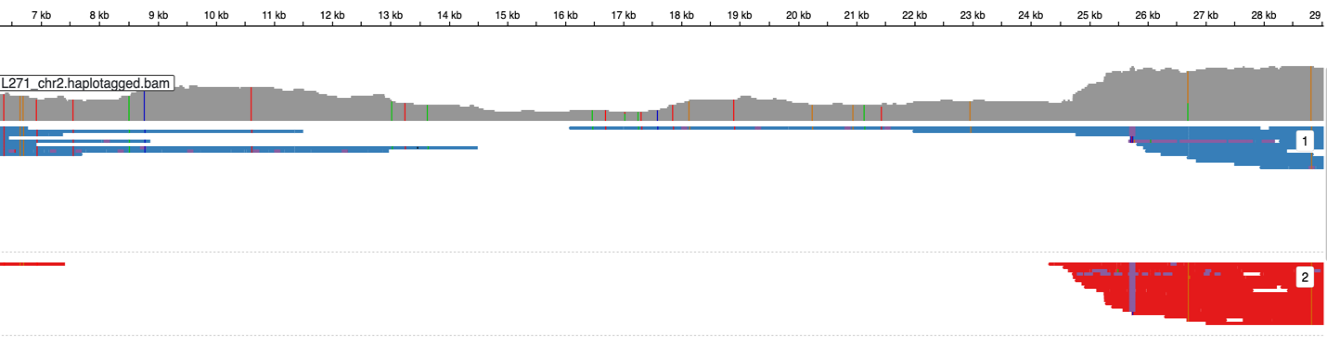


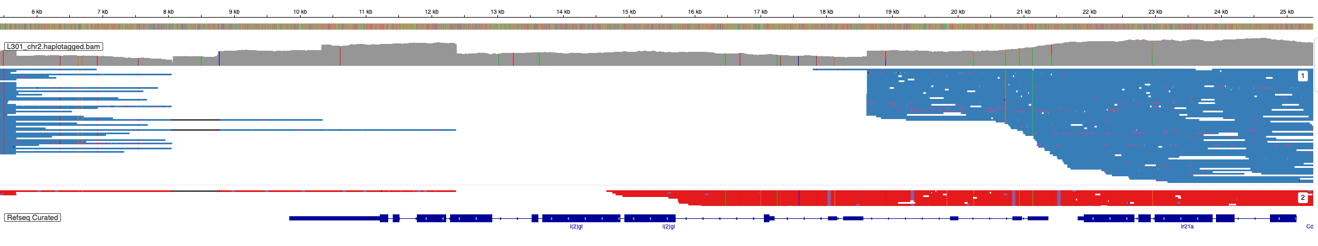


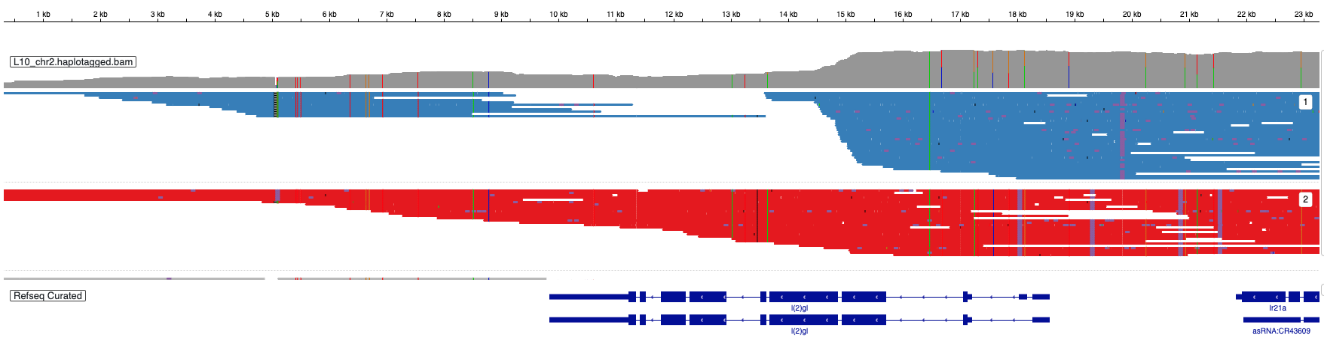


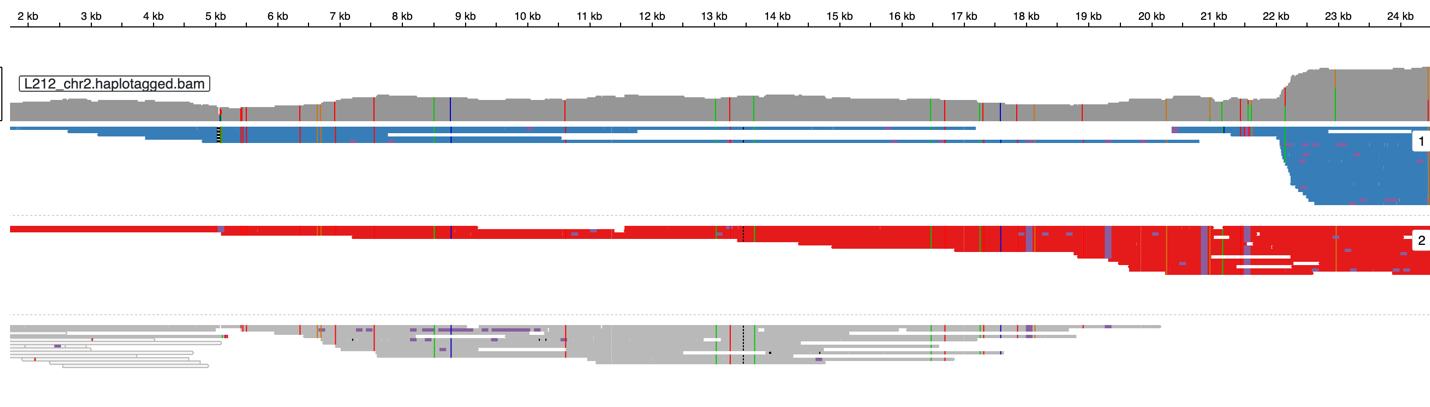

Supplement: S1 Fig — The following images show the PacBio Revio reads aligned to the distal end of 2L. The two colors in the view of each subline depict reads phased to different haplotypes by HiPhase. (DOCX) [file pbio.3003467.s004.docx]
