# Supplementary material for: Transposable elements contribute substantially to naturally occurring genetic lethality in Drosophila melanogaster
Source: PLoS Biol. 2026 Mar 10;24(3):e3003467. doi: 10.1371/journal.pbio.3003467 (PMC12974806; doi:10.1371/journal.pbio.3003467)
Supplement: Attachment — Submitted filename: Response to Reviewers.docx [file pbio.3003467.s012.docx]

**Response to Reviewers**

*Please note that line numbers refer to the line numbers in the track-changes revision document, opened in Microsoft Word, with “Simple Markup” selected.*

REVIEWS:

Reviewer #1: The authors mapped recessive deleterious mutations from a natural D. melanogaster population and found that many mutations were caused by insertions of two TEs, which recently invaded D. melanogaster.
The study is thoroughly executed and the results are interesting. Nevertheless, I do not agree with the interpretation of the authors:

1) It was recently shown that variable strength of purifying selection could generate the same pattern as insertion bias (Langmuller et al. NAR 2022)- insertions are preferentially detected at specific genomic positions. Hence, it is conceivable that the genes with recessive lethal TE insertions are not very strongly counter selected because they were highly recessive. This interpretation would not only be supported by the presence of multiple independent insertions at the same gene, but also by the observation of the authors that the same gene was hit by another mutation causing a recessive deleterious allele (Nipped-A and CG33155). Hence, the results would be mostly affected by the dominance of a gene and the most recessive genes would be enriched for recessive lethals. While the reviewer considers this the most plausible interpretation of the data, the reviewer accepts the speculative nature, but requests that the hypothesis is adequately discussed in a revised manuscript.

***Author response****: This is an interesting paper and I appreciate that you bring up the potential variation in recessivity / dominance coefficients of the lethal mutations, which have been found to show some variation in historical studies of lethal chromosomes. In the discussion, I have now referenced the Langmüller et al paper and included the caveat on the insertion site preference hypothesis, that the sites with high frequencies of lethal TE insertions could instead be the result of weak selection at these sites or alleles (one reason being that they those are the most highly recessive) (tracked changes version lines 508-510). We now emphasize that our paper is studying lethal mutations currently segregating in natural populations, and not all lethal mutations that initially appear in populations (because those that originally appear but are more dominant may be selected out extremely quickly).*

2) If the large number of independent mutations at some of the genes does not reflect an insertion bias, but differential selection, this implies that the TE invasion has resulted in many mutations of the same gene. In this case, the reviewer is not clear about the expectations to detect point mutations or TE insertions to cause lethal mutations. Maybe, this could be modelled by comparing a mutational burst to continuously emerging base substitutions. After all, the number of genes affected by base substitutions is not so small.

***Author response****: It is true that our results are correlative and we do not directly test causation of each candidate lethal mutation. We do not wish to claim definitively that the best ‘candidate lethal mutations’ must be the cause of lethality, rather that their locations match with where we have fine mapped the genetic basis of lethality. I have added this caveat on tracked changes version lines 649-651. We have confidence in the ability to detect both point mutations and TE insertions, given PacBio Revio long read sequencing and detection using both computational variant-calling tools specified for different mutation types and careful manual scanning and detection using sequence read visualizers (such as IGV). All variants (SNPs, etc) detected were then characterized by probable effect size using SnpEff, to help filter out low impact, silent mutations. There are many single base variants found along the genome and within genes with known lethal effects, but in the regions with known lethal* ***mutations, there were no single nucleotide variants (except those in drosha) identified that were predicted to have high effect****, and most were silent mutations and in introns. The transposable element insertions were the leading candidates to have lethal effects in the other regions of interest. While not definitive evidence (e.g., the algorithm is almost definitely imperfect), the observed mutations are indeed likely strong candidates, though again, we appreciate the need for adding the caveat.*

Apart from the interpretation of the data, the reviewer would like to see additional evidence for the heterogeneity in TE insertion positions. Mapping reads with TE insertions to a reference genome without them is clearly challenging. Hence, the reviewer would like to see an alignment of reads spanning the insertions.

***Author response****: Thank you for pointing this out! I have now added TE read alignments and consensus sequences in the supplemental material.*

Minor points:
Isoline seems to be internal lab slang-the reviewer prefers the term isofemale line. Wikipedia thinks that an isoline is something entirely different…

***Author response****: This is a good point, thank you! We do not use the phrase isofemale line because the balanced lab stocks derived from a single wild fly and a balancer line are not technically isofemale lines, and because most of the wild flies were male. However, I agree that this terminology of ‘isoline’ is confusing and have removed it in all instances. The paper now only refers to ‘lethal lines’ which is defined on tracked changes version lines 181-183.*

Reviewer #2: The study investigates the long-standing question of why recessive lethal alleles persist at unexpectedly high frequencies in natural population of Drosophila melanogaster. By combining classical balancer-based complementation mapping with whole genome sequencing of 293 wild second chromosome lines, the authors demonstrate that most lethals seem single-locus, loss-of-function mutations and frequently arise independently in the same 'essential genes.' Interestingly, they find that approximately 80-90% of these mutations occur in relation to transposable element (TE) insertions. The authors propose that recurrent TE insertions, with their much higher mutation rates than single-nucleotide changes, can account for the observed frequencies of lethal alleles through mutation-selection balance. They further suggest that recent TE invasions create transient bursts of lethality that are later purged as host suppression evolves.
Overall, the manuscript reframes the evolutionary origin of natural lethality as a dynamic consequence of TE activity. However, there are some concerns that should be addressed to be sure that the conclusions are supported by the presented data.

Major Comments:

1) In general, the authors exclude alternative hypotheses to explain their data with insufficient support.

a. In the introduction the authors heavily focus on mutation-selection balance and continue to build their arguments on its inadequacies to explain the observed higher frequencies of lethal mutations present. Even though they briefly mention balancing selection (both in the introduction and discussion) they dismiss it and it is not given enough attention to rigorously test. This is also reflected in that they do not test for or present explanations for any heterozygote fitness that might be in play.

***Author response****: This is a great point and I did not intend to leave out discussions of balancing selection in the introduction or the discussion. Thanks! Previous tests for heterozygote advantage have not revealed much evidence for it. However, they were usually lab fitness assays, which are limited to lab environments and not indicative of natural conditions and did not explore other forms of balancing selection (frequency dependent selection, or associative overdominance are other forms that I have theoretically considered and have a separate manuscript in the works modelling potential effects of linked selection and associative overdominance on lethal mutation frequencies). My results, given that most all ‘high frequency’ lethal allelic sets were due to mutations that are not identical by descent, did not suggest likely balancing selection. I have now added reference to previous studies attempting to detect heterozygote advantage of naturally occurring lethal mutations in the introduction (tracked changes version lines 134-139) and please refer to tracked changes version lines 500-508 for explanation as to why we do not find evidence supporting the effort and expense of additional tests for balancing selection. I also suggest future work to explore the potential heterozygote advantage of the lethal telomeric deletion found in 9 different lethal lines (tracked changes version lines 604-606).*

b. In addition, explanations other than TE insertions (e.g. other structural variation, demography, genetic drift) also need to be evaluated as the manuscript largely leads the reader to believe TEs are not only the major source but almost the sole driver of lethal mutations.

***Author response:*** *I agree! I don’t want to lead the reader to believe that TEs are the sole drivers of lethal mutations, and I hope that my revisions have clarified that message. For the lethal effects mapped in this study, the best candidate mutations are discussed for each case, many of which happen to be transposable element insertions. I have added in the discussion that this is a small sample of lethal mutations from a single population of one species and this does not mean that all lethal mutations are the result of transposable element insertions (tracked changes version lines 645-653). I completely agree that naturally occurring lethal effects, as most things in nature, cannot be fully explained by one cause. Undoubtedly, many lethal mutations exist in nature due to other mutation types or structural variants and could be maintained by numerous evolutionary forces including drift (population bottlenecks or seasonal population size fluctuation), balancing selection or linked selection. The intersection of these is not explored in this study as our results did not lend to those analyses.*

c. The authors should be careful when interpreting results: Failure to reject the null hypothesis is not proof of the null hypothesis being true (type I vs type II errors are different) (see lines 256-266). The conclusions should be toned down, or the null hypothesis reframed so the authors can actually perform a test to see if their hypothesis (the non null) is more likely.

***Author response****: Yes, agreed, thank you for pointing this out. I have revised the statement (tracked changes version lines 245-256).*

2) Several strong claims are made without direct evidentiary support and should be toned down or removed if the authors do not perform additional validation analyses/experiments.

***Author response****: Thank you. See my responses to each point individually and corresponding edits made in the manuscript in response.*

a. The claim that TE mutation rates (not only invasion) explain high lethal frequencies is not backed by quantitative parameters.

***Author response****: Agreed. I have added to the discussion previously estimated TE mobilization rates, contrasted them to point mutation rates and discussed many factors contributing to the challenge of quantitative parameters around TE rate mobilization (lines 620-631). TE mobilization rates estimates are limited due to recency of interest in TE rates (relative to other mutation types) and difficulty of detection, rates differ between TE type and recency of invasion to the host species and we are lacking a standard unit of measure for TE mobilization rates none of which are easily comparable to other mutation rate units (number of events per chromosome, number of transpositions per element, etc). Furthermore, because Transib1 and Kuruka were only discovered in D. melanogaster within the last few years, there are no published estimates on their mobilization rates or insertion site preference. Our whole genome sequence data could be used to investigate these questions in a future study.*

b. The finding that the majority of mapped lethal alleles coincides with TE insertions is not supported by functional data to confirm these events are indeed causal, with mere correlative evidence to support it. Thus, the statement "TEs drive much of naturally occurring lethality" goes beyond what the data shows.

***Author response****: The reviewer is correct that we did not do functional experiments inserting or removing the transposable elements observed to confirm their effects. While such a major disruption of an essential gene can be generally anticipated to cause lethality, the reviewer is correct that the results presented are formally correlative, and I thus say this explicitly now (lines 649-651). Good call.*

3) The manuscripts central claim that high lethal mutation frequencies can be explained by mutation-selection balance (a long-term equilibrium) once TE mutations are considered presents an inconsistency with the interpretation of the findings that these TE invasions are transient. If said invasions are dynamic and result in short-term elevations which would be selected out once the species develops a silencing mechanism for the invader then the hypothesis that it is a long-term equilibrium wouldn't stand. This should be evaluated and discussed.

***Author response****: Great point and I agree- mutation-selection balance is not the appropriate term for the proposed model, as it predicts transient ‘lethal hotspots’ that fluctuate in frequency and location. I have revised the phrasing and discussion the potential hypotheses that recent TE invasions could result in new ‘lethal hotspots.’ (lines 611-617).*

4) The authors report several allelic sets that show high kinship (likely due to sampling bias) but do not mention any correction for it.

***Author response****: Thank you. The primary allelic set that has high kinship values between individuals was the set that mapped to the gene, drosha. We discuss that this set of identical lethal mutations is due to sampling bias (of related individuals) on lines 316-322. Elevated kinship coefficients were also present between three of the 4 lethal lines that carry a potentially lethal insertion in CG33155, and we discuss in the results that they are related (lines 363-366). The two instances in our data of lethal mutations that are identical by descent is likely explained by sampling bias, and it is further evidence that there is little reason to believe that balancing selection explains most of the findings in our data (see response to 1a).*

5) The authors introduce 'Kuruka' as a novel Gypsy-like retrotransposon implicated in several mapped lethal alleles. Although this could be an exciting discovery, more information is required in this case, including a consensus sequence and genomic coordinates for insertions, along with phylogenic comparison with other established Gypsy-like elements, as well as its distribution in other (lab or not) D.melanogaster populations.

***Author response****: Agreed! In fact, I was just waiting for Riccardo Pianezza to submit his manuscript so that I could site it officially (and not give away his results). Please see lines 592-601 for updated information and citations regarding Kuruka and additional supplemental material I have added with consensus TE sequences.*
