# Supplementary material for: Transposable elements contribute substantially to naturally occurring genetic lethality in Drosophila melanogaster
Source: PLoS Biol. 2026 Mar 10;24(3):e3003467. doi: 10.1371/journal.pbio.3003467 (PMC12974806; doi:10.1371/journal.pbio.3003467)
Supplement: Attachment — Submitted filename: Response to Reviewers_v2.docx [file pbio.3003467.s013.docx]

**Response to Reviewers, version 2**

*Please note that line numbers refer to the line numbers in the track-changes revision document, opened in Microsoft Word, with “Simple Markup” selected.*

REVIEWS:

Reviewer #1: The authors have responded to my comments, but prefer to stick to their original interpretation. I think this is perfectly ok, as they at least discuss alternative interpretations.

My only concern that remains is their weak justification that SNPs are not causative. It is well-understood that even silent mutations can have large fitness consequences-there is a large body of literature on this in bacteria. Along the same lines-intronic sequences are often very important for gene regulation. Hence, I think that some more careful phrasing is required for this aspect of the manuscript as well.

***Author response****: Thank you for emphasizing this point! I agree that we do not prove that SNPs are not causative and we cannot rely on predicted effect size as an exact proxy for phenotypic impact – there are very interesting examples exist of highly deleterious silent or intronic SNPs in nature. We focus on the fact that there are no shared SNPs within lethal sets that are unique to those sets (accept the one noted in Drosha), and that the TE insertions* ***are*** *shared within sets and unique to each (tracked changes line 654-656). I try to highlight in the paper that our results are correlative, so we can’t know for certain from our data alone, but that the results of our study warrant further investigation into lethal effects of transposable elements.*

Reviewer #2: The authors have carefully responded to my previous concerns. I am satisfied that the presented conclusions are supported by the data and analyses.
